# Impaired Gut Epithelial Tight Junction Expression in Hemodialysis Patients Complicated with Intradialytic Hypotension

**DOI:** 10.1155/2018/2670312

**Published:** 2018-01-16

**Authors:** Tsai-Kun Wu, Paik-Seong Lim, Jong-Shiaw Jin, Ming-Ying Wu, Chang-Hsu Chen

**Affiliations:** ^1^Division of Renal Medicine, Tungs' Metroharbor Hospital, Taichung, Taiwan; ^2^Department of Rehabilitation, Jenteh Junior College of Medicine, Nursing and Management, Miaoli, Taiwan; ^3^Department of Pathology, Tungs' Metroharbor Hospital, Taichung, Taiwan

## Abstract

**Background:**

There is accumulating evidence pointing to uremia-induced impairment of the intestinal epithelial barrier structure in advanced chronic kidney disease (CKD) and hemodialysis (HD) patients. In this study, the impact of intradialytic hypotension on intestinal barrier integrity is being explored.

**Methods:**

Immunohistochemical staining was used to detect the expression of 4 types of tight junction (TJ) proteins such as occludin, zonula occludens-1 (ZO-1), claudin-1, and claudin-4, in colonic samples of a group of patients receiving segmental colectomy. Five patients with nondialysis CKD (group 2), 5 HD patients with intradialytic hypotension (group 3), and 5 non-CKD subjects (group 1) were examined.

**Results:**

Both patients' groups 2 and 3 demonstrated significantly reduced expression of occludin as compared to group 1 (*p* < 0.05 and *p* < 0.01, resp.). Except for claudin-4, expression of all markers of TJ proteins was significantly reduced in patients' group 3 as compared to control (*p* < 0.01). In addition, decreased expressions of claudin-1 and ZO-1 were also more pronounced in group 3 when compared to group 2.

**Conclusions:**

This study extends the earlier finding by demonstrating that dialysis-related hypotension caused even marked depletion of the key protein constituents of the epithelial TJ.

## 1. Background

Chronic inflammation is a common and notorious feature in patients with chronic kidney disease (CKD). Besides, it is a good predictor for cardiovascular mortality as well as many adverse complications in these patients [1]. The gastrointestinal mucosa forms a barrier between the body and a luminal environment. Disruption of barrier integrity may be responsible for the entry of hostile microorganisms and toxins, leading to various inflammatory syndromes. To defend aganist the entry of microbes, harmful toxin, and antigen, gastrointestinal (GI) epithelial tight junction (TJ) serves as an important barrier. So, reduced levels of expression of TJ may lead to impaired epithelial barriers and loss of protection, leading to entry of intestinal microbes and resulting in inflammation. In recent few years, intestinal barrier dysfunction in uremic animals or human has been increasingly reported [2, 3].

Some previous studies suggested that circulating endotoxin levels are higher in patients with advanced CKD and increased with worsening of renal function [4, 5]. In fact, hemodialysis (HD) initiation itself was significant associated with a higher endotoxemia. Moreover, in HD patients, predialysis endotoxin correlated with dialysis-induced hemodynamic stress, including relative hypotension [4].

The TJs are the apical most constituent of the apical junctional complex in epithelial cell sheets. TJs are composed of transmembrane proteins, such as occludin and members of the claudin family, and cytoplasmic plaque proteins, including the zonula occludens (ZO-1) proteins, that link the transmembrane proteins to the actin cytoskeleton [6, 7]. TJs are regulated in their molecular composition, ultrastructure, and function by intracellular scaffolding proteins and the cytoskeleton. TJs create the major barrier regulating paracellular movement of water and solutes across epithelia. In addition, TJs form the continuous intercellular barrier between epithelial cells, which is required to prevent the entry of potentially harmful microbes, toxins, and antigens from the intestinal lumen. Reduced TJs integrity greatly increases ion conductance across the paracellular route compared to the transcellular route, facilitating the access of pathogens and endotoxins. While TJs require the coordinated activity of several different proteins, the specificity of TJ permeability is regulated by claudins. There is accumulating evidence that claudins constitute the backbone of TJs strands and are responsible for the regulation of paracellular selectivity to small ions. On the other hand, occludin, the first TJ-specific integral membrane protein identified, contributes to TJ stabilization and optimal barrier function [8]. Unlike the above proteins, ZO-1 protein family is a kind of cytosolic protein to bind the other protein such as occludin, claudin, and the perijunctional actin-myosin ring and as such serves an essential role in the TJ assembly and function [9].

Intradialytic hypotension (IDH), a common complication in hemodialysis patients, impairs patients' quality of life by causing nuance symptoms and creates barriers to achieving adequate dialysis dose and ultrafiltration [10]. The National Kidney Foundation Kidney Disease Outcomes Quality Initiative (KDOQI) defines intradialytic hypotension (IDH) as a decrease in systolic blood pressure by ≥20 mm Hg or a decrease in MAP by 10 mm Hg associated with symptoms [11]. IDH can induce cardiovascular complications, including cardiac arrhythmias and coronary and cerebral ischemic event and in the long term it may lead to higher hospitalization and mortality rate [10, 12, 13]. Moreover, emerging evidence showed that regional ischemia and HD-associated circulatory oxidative stress worsened and may lead to endotoxin translocation from the gut [4, 12].

In order to elucidate the relationship between gut TJ, IHD, and CKD, we hypothesize that the expression of TJ proteins, which regulate gut paracellular permeability, is altered in the intestinal mucosa of patients with CKD. In addition, the potential role of intradialytic hypotension is also being examined in CKD patients receiving HD.

## 2. Methods

This retrospective study enrolled patients had undergone colectomy from Tungs' Metroharbor Hospital between the year 2008 and 2013. A total of 8 dialysis patients and 7 nonuremic controls undergone segmental colectomy were enrolled. The age of the patients varied from 44 to 90 years for both men and women. Control samples were tissue resected during surgical intervention for various causes as shown in Table 1. Formalin-fixed, paraffin-embedded intestinal tissue blocks of these patients were retrieved from the files of the Department of Pathology of Tungs' Taichung Metroharbor Hospital. These 15 patients were divided into 3 groups, including 5 patients without ischemic bowel disease or CKD (group 1 also as controls), 5 advanced CKD patients without ischemic bowel disease (IBD) (group 2), and 5 hemodialysis patients with intradialytic hypotension complicated with IBD (group 3). Patients on group 3 suffered from at least 3 times of IDH a month. All studied tissue samples were obtained from patients under protocols approved by Institution Review Board of Tungs' Metroharbor Hospital (TTMHH number 102046). All participants gave written informed consent.

### 2.1. Histological and Immunohistochemical Procedures

Each paraffin-embedded tissue was reviewed by pathologists and cut into 4-micrometer tissue slides and stained with haematoxylin and eosin (H&E) or immunohistochemistry stains.

Claudin-1 antibody, claudin-4 antibody, occludin antibody, and Zona occludens-1 (ZO-1) antibody were purchased from Biorbyt Company (Cambridge, Cambridgeshire, United Kingdom). Briefly, the tissue slide sections were dewaxed in xylene, rehydrated in alcohol, and immersed in 3% hydrogen peroxide for 5 minutes to suppress endogenous peroxidase activity. Antigen retrieval was performed by heating (100°C) each section for 30 minutes in 0.01 mol/L sodium citrate buffer (pH 6.0). After 3 rinses (each for 5 minutes in phosphate buffered saline [PBS]), sections were incubated for 1 hour at room temperature with a claudin-1 antibody, claudin-4 antibody, occludin antibody, or ZO-1 antibody diluted in PBS as previous studies [14–16]. After 3 washes (each for 5 minutes in PBS), sections were incubated with biotin-labeled secondary immunoglobulin (1 : 100, DAKO, Glostrup, Denmark) for 1 hour at room temperature. After 3 additional washes, peroxidase activity was developed with DAB (DAKO, Glostrup, Denmark) at room temperature.

Staining intensity of all immunostains was scored semiquantitatively by two separate observers. The scoring system used was in accordance with earlier publications [14] on intensity of staining as well as number of stained cells, with intensity scores of 0 (no staining) to 4 (strongest intensity), and the percentage of stained cells was estimated at each intensity. The percentage of cells (from 0 to 100) was multiplied by the corresponding immunostaining intensity (from 0 to 4) to obtain immunostaining scores ranging from 0 to 400. Normal colonic epithelium served as an external positive control for all antibodies and showed expression of all proteins in a particulate pattern, confined to the apical portion of the lateral epithelial cell membrane.

### 2.2. Statistical Analysis

All results are expressed as mean ± standard error of the mean (SEM). The SEM was analyzed on the various cases of the same group. The immunostaining scores of claudin-1, claudin-4, occludin, and ZO-1 in colonic tissues were compared among these groups (group 1 as normal epithelia). Statistical analysis was performed using the Mann–Whitney *U* test and Kruskal-Wallis test between groups and a *p* value of less than 0.05 was considered statistically significant.

## 3. Results

### 3.1. Clinicopathological Characteristics

**Figure 1:**
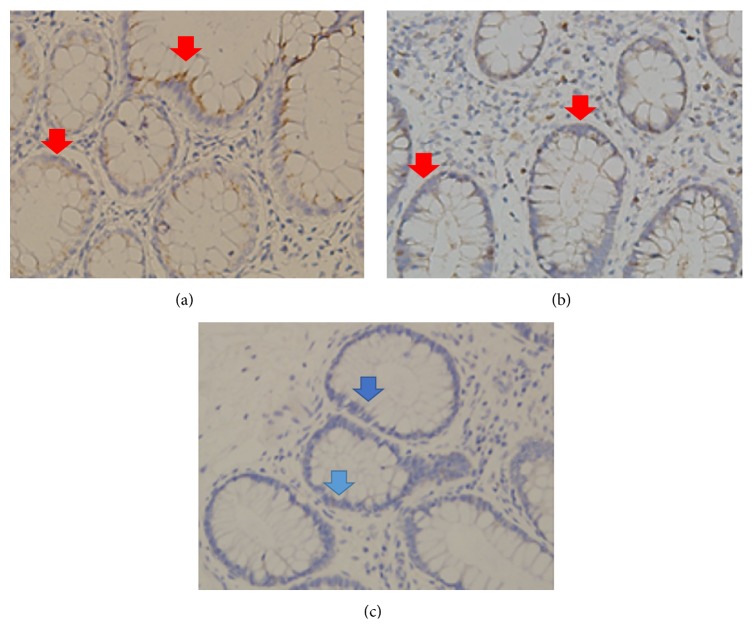
Immunohistochemical expression of claudin-1 in the colon mucosa of patients with non-CKD (control) (a) or advanced CKD (b) HD patients with hypotension (c). There was no significant difference in claudin-1 expression between non-CKD and CKD patients (red arrows). A significant decrease in claudin-1 staining extent and intensity was only seen in HD patients (blue arrows) (c).

**Figure 2:**
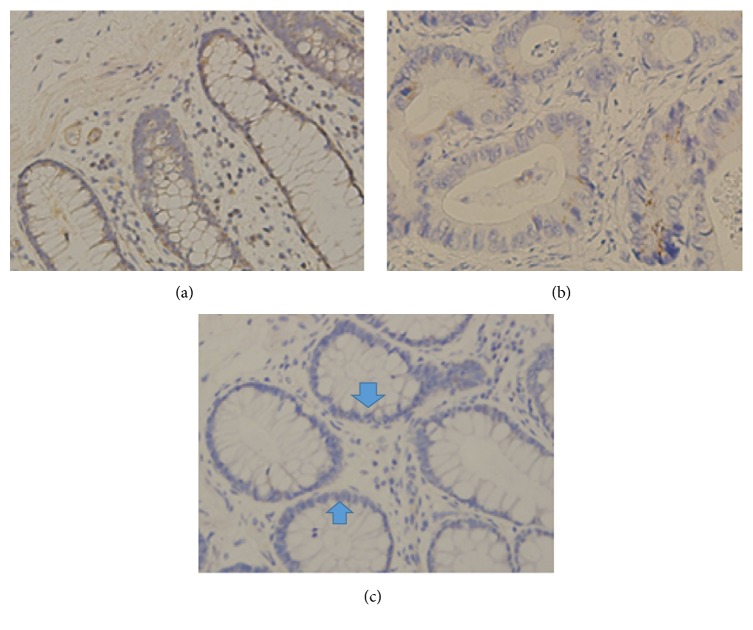
Immunohistochemical expression of occludin in the colonic mucosa of patients with (a) non-CKD, (b) advanced CKD, and (c) HD with hypotension. The total of epithelial cells lining villi exhibited positive immunostaining for occludin in healthy controls (a). In patients with advanced CKD, occludin expression is reduced in numerous epithelial cells (b). In HD patients with IDH, there was profound loss of occludin expression by most epithelial cells lining villi (blue arrows) (c).

Characteristics of the enrolled patients are summarized in Table 1. Their age ranged from 44 to 90 years (70.4 ± 14.1) and there were fourteen males and only one female. All control tissues were obtained from patients without CKD. Specimens from controls were distributed throughout colon and there was no apparent association between location and tight junction protein expression. Differential expression of claudin-1, claudin-4, occludin, and ZO-1 of the three groups are shown in Figures 1 and 2.

### 3.2. Colonic TJ Proteins Immunohistochemical Results of the 3 Groups

**Figure 3:**
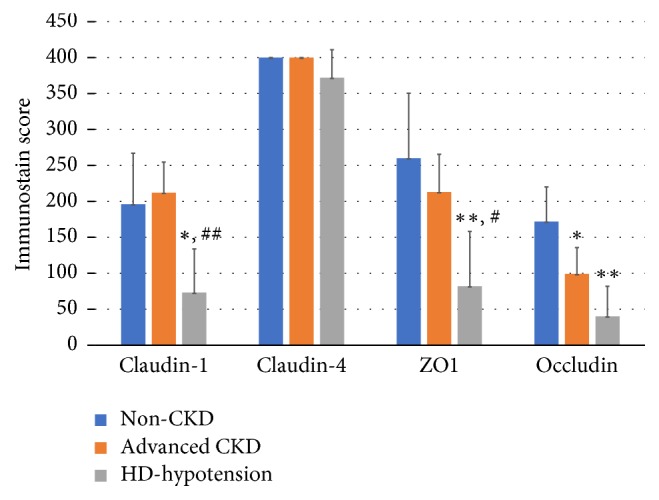
Immunostaining scores among nonchronic kidney disease (non-CKD; controls), advanced chronic kidney disease (advanced CKD), and hemodialysis patients complicated with hypotension (HD-hypotension). *∗* indicates *p* < 0.05 compared to non-CKD; *∗∗* indicates *p* < 0.01; # indicates *p* < 0.05 compared to non-CKD; ## indicates *p* < 0.01

The basic characteristics and immunostaining scores among 3 groups are summarized in Table 2 and Figure 3. In patients of group 3, there was a significant decrease in staining extent and intensity of claudin-1, occludin, and ZO-1 as compared to group 1. The semiquantitative analysis of TJ proteins immunohistochemical results also showed that group 3 of patients presented significantly reduced expression of occludin, ZO-1, and claudin-1 as compared to patients in group 2 (*p* < 0.001, *p* < 0.05, *p* < 0.05, resp.). However, compared to group 1, only occludin expression was significantly reduced in group 2 of patients. More interestingly, expression of occludin gradually decreased with increasing severity of renal dysfunction as shown in Table 2.

On the other hand, claudin-4 expression was not significantly different in tissues of both groups 2 and 3 as compared to control tissues.

## 4. Discussion

In this study, we showed that there is marked decreased expression of some TJ-associated proteins in hemodialysis patients complicated with hypotension when compared to the non-CKD control group. TJs are multiprotein complexes which are crucial for the integrity and function of the epithelial barrier. Downregulated expression of intestinal TJ proteins may result in loss of intestinal barrier function and altered mucosal permeability. Loss of barrier integrity may in turn facilitate translocation of microbes and its metabolites that may result in increased susceptibility to systemic infection and chronic inflammation. To the best of our knowledge, this study demonstrates for the first time the markedly decreased expression of some TJ-associated proteins in the intestinal epithelium of hemodialysis patients suffering from repeated intradialytic hypotension.

Increased intestinal permeability has been demonstrated in a number of experimental CKD and the clinical settings [2, 4, 17, 18]. In animal models of CKD, depletion of some the key protein constituents of intestinal epithelial TJ proteins, mostly claudin-1, occludin, and ZO-1 [17], was found. Retained uremic toxins were reported as one of the major causes of impaired intestinal epithelial barrier. Vaziri et al. [18] found that addition of collected plasma from patients with end-stage renal disease results in marked depletion of TJ proteins in cultured human enterocytes. However, in our study, compared to non-CKD patients, in patients with advanced CKD (average creatinine was 9.0 ± 5.5 mg/dl), downregulation of claudin-1, claudin-4, and ZO-1 expression was not observed. Only the occludin protein expression was significantly reduced in patients with advanced CKD. Though these patients are exposed to marked noxious uremic environment for extended periods of time, the expression of some of the TJ proteins is only modestly affected. This observation seems to suggest that probably uremic milieu per se may not be the sole cause of the degradation of epithelial TJ proteins in CKD patients. Other factors like ischemia with hypoxic injury may work in concert with causing disruption of the intestinal barrier. However, reduced expression of some TJ-associated protein such as occludin appeared to occur at earlier stage of CKD as demonstrated by the results of immunostaining scoring assessment. This finding can be partly explained by the fact that the occludin may be more vulnerable to disruption by some uremic toxins. Only after repeated ischemic injury such as intradialytic associated hypotension, the intestinal epithelial integrity may become more widespreadly disrupted. Interestingly, McIntyre et al. found that significant incremental endotoxemia across the spectrum of CKD, with levels 5-fold higher in patients on dialysis compared to predialysis CKD stage 5 [4]. Moreover, they also demonstrated significant correlation between serum endotoxin and intradialytic instability and risk of subsequent mortality [4]. Accumulating evidence suggested that IDH was associated with cardiovascular morbidity and mortality [12, 19]. HD-induced systemic circulatory stress and recurrent regional ischemia may lead to increased endotoxin translocation from the gut [4]. Earlier studies had reported that ultrafiltration causes a reduction in splanchnic blood volume in HD patients [20] even when their arterial blood pressure is preserved [21]. Mesenteric ischemia can result in disruption of gut mucosal structure and function, with increased gut permeability as well as increased microbial and its metabolites translocation [22]. Intradialytic hypotension during HD may further aggravate regional hypoperfusion and mesenteric injury. In our study, compared to advanced CKD patient, we found that HD patients with recurrent intradialytic hypotension have more markedly reduced expression of some of their TJ proteins. Moreover, one recent study [23] showed that more frequent HD regimen is associated with lower levels of circulating endotoxin compared with conventional HD. Presumably, better hemodynamic stability with this dialysis modality allows superior maintenance of regional splanchnic perfusion and less extensive damage to the intestinal mucosa. Though the precise underlying mechanism of our observation required further studies, clearly our finding suggested that avoidance of repeated intradialytic hypotension is one of the rational approaches for protecting the integrity of the intestinal barrier.

Despite these interesting findings, our study has a number of limitations, including its retrospective nature, a small sample size, and the fact that this study was conducted in a single institution. The small sample size limits the power of the study and may have prevented the detection of some differences among study groups. Besides, the limited number of samples did not allow us to identify the progressive changes in TJ proteins in different stages of CKD.

## 5. Conclusion

Reduced expression of some intestinal epithelial TJ proteins was observed in HD patients. IDH may be an important aggravating factor disrupting the intestinal barrier integrity in these patients. Further studies are needed to fully establish the mechanisms underlying the pathophysiology of impaired gut barrier in HD patients.

## Figures and Tables

**Table 1:** Basic characteristics of the patients.

Number	Status	M/F	Age	IDH	DM	Types of surgery
1	None	M	82	N	Y	Sigmoid diverticulitis
2	None	M	44	N	Y	Redundancy
3	None	M	75	N	N	Perforated diverticulitis with peritonitis
4	None	M	55	N	Y	Perforation with abscess
5	None	M	74	N	N	Hyperplastic polyp
6	CKD4	M	81	N	N	Hartmann's operation, Sigmoid volvulus
7	CKD4	M	64	N	Y	Diverticulitis with granuloma
8	CKD5	M	83	N	Y	Colon obstruction
9	CKD5	M	90	N	N	Right hemicolectomy, tumor of cecum, A colon perforation
10	CKD5	M	59	N	Y	Diverticulitis
11	HD	M	83	Y	Y	Ischemia, diverticulosis with perforation
12	HD	M	71	Y	Y	Ischemic bowel
13	HD	M	75	Y	N	Ischemic bowel necrosis of terminal ileus and ascending colon
14	HD	M	45	Y	N	Perforation and ischemic bowel
15	HD	F	75	Y	N	Perforation and ischemia

M: male; F: female; CKD: chronic kidney disease stage; HD: hemodialysis; IDH: intradialytic hypotension; DM: diabetes mellitus; N: none; Y: yes.

**Table 2:** Immunostaining scores and basic characteristics among three groups of studied patients.

	Non-CKD (*n* = 5)	Nondialysis CKD (*n* = 5)	HD-hypotension (*n* = 5)
Age	66.2 ± 16.0	74.0 ± 12.9	69.8 ± 14.5
M/F	5/0	5/0	4/1
Serum Cr	1.1 ± 0.2	9.0 ± 5.5^*∗*^	10.2 ± 2.3^*∗∗*^
TJ protein			
Claudin-1	196.0 ± 70.9	212.0 ± 42.7	73.0 ± 60.8^*∗*,##^
Claudin-4	400.0 ± 0.0	400.0 ± 0.0	372.0 ± 39.0
ZO-1	260.0 ± 90.3	213.0 ± 52.6	82.0 ± 76.3^*∗∗*,#^
Occludin	172.0 ± 48.2	99.0 ± 36.8^*∗*^	40.0 ± 42.0^*∗∗*^

M: male; F: female; CKD: chronic kidney disease stage; HD: hemodialysis; IDH: intradialytic hypotension; Cr: creatinine; TJ: tight junction. Compared to group 1, ^*∗*^*p* < 0.05, ^*∗∗*^*p* < 0.01; compared to group 2, ^#^*p* < 0.05, ^##^*p* < 0.01.

## Abbreviations

CKD: Chronic kidney disease
GI: Gastrointestinal
TJ: Tight junction
HD: Hemodialysis
ZO: ZONULA occludens
KDOQI: Kidney Foundation Kidney Disease Outcomes Quality Initiative
IHD: Intradialytic hypotension
H&E: Haematoxylin and eosin
PBS: Phosphate buffered saline
SEM: Standard error of the mean
ESRD: End-stage renal disease.
